# Assessment of intracardiac and extracardiac anomalies associated with coarctation of aorta and interrupted aortic arch using dual-source computed tomography

**DOI:** 10.1038/s41598-019-47136-1

**Published:** 2019-08-12

**Authors:** Qin Zhao, Jin Wang, Zhi-gang Yang, Ke Shi, Kai-yue Diao, Shan Huang, Meng-ting Shen, Ying-kun Guo

**Affiliations:** 1 Department of Radiology, West China Hospital, Sichuan University, 37# Guo Xue Xiang, Chengdu, Sichuan 610041 China; 2 Department of Radiology, West China Second University Hospital, Sichuan University, 20# Section 3 South Renmin Road, Chengdu, Sichuan 610041 China

**Keywords:** 3-D reconstruction, Anatomy, Congenital heart defects, Aortic diseases, Congenital heart defects

## Abstract

To evaluate the value of dual-source computed tomography (DSCT) compared with transthoracic echocardiography (TTE) in assessing intracardiac and extracardiac anomalies in patients with coarctation of aorta (CoA) and interrupted aortic arch (IAA). Seventy-five patients (63 with CoA and 12 with IAA) who received preoperative DSCT and TTE were retrospectively studied. Intracardiac and extracardiac anomalies were recorded and compared by DSCT and TTE, in reference to surgical or cardiac catheterization findings. A total of 155 associated anomalies were finally found. Collateral circulation (56, 74.70%), patent ductus arteriosus (PDA; 41, 54.67%) were the most common anomalies. PDA, aortopulmonary window, and collateral circulation were more frequently present in patients with IAA than those with CoA (100% vs. 46.03%, 16.67% vs. 0%, and 100% vs. 69.84%, respectively, all *p* < 0.05). DSCT was superior to TTE in assessing associated extracardiac-vascular anomalies (sensitivity: 100% vs. 39.81%; specificity: 100% vs. 100%; positive predictive value: 100% vs. 100%; negative predictive value: 100% vs. 76.06%). Extracardiac-vascular anomalies, including collateral circulation and PDA, were the most common anomalies in patients with IAA and CoA. Compared with TTE, DSCT is more reliable in providing an overall preoperative evaluation of morphological features and extracardiac anomalies for surgical planning.

## Introduction

Coarctation of aorta (CoA) and interrupted aortic arch (IAA), manifested as obstructive anomalies of the aortic arch, are rare congenital heart diseases associated with aortic arch developmental disorders^1^. Patent ductus arteriosus (PDA), ventricular septal defect (VSD), bicuspid aortic valve (BAV), and many other intracardiac and extracardiac anomalies have been reported by previous studies in patients with CoA and IAA^2,3^. Combined complex anomalies, especially extracardiac defects and collateral circulation, play an important role in the systemic hemodynamic changes and surgical or interventional strategy-making^1,2,4^. Thus, rapid and accurate morphological diagnosis and evaluation of the two aortic arch defects and associated malformations are necessary to achieve a comprehensive assessment for treatment planning.

In recent years, computed tomography (CT), especially dual-source computed tomography (DSCT), has been used widely in the evaluation of congenital heart diseases as a noninvasive imaging technology, and it is considered a reliable method to assess great vessels^5–8^. To the best of our knowledge, there are relatively few studies using DSCT for comprehensively preoperative evaluation of CoA, IAA, and associated malformations, and IAA is always showed as case report^9–12^. Therefore, we aimed to identify the overall value of DSCT compared with transthoracic echocardiography (TTE) in evaluating the morphological characteristics of aorta and the associated intracardiac and extracardiac malformations in patients with unrepaired CoA and IAA.

## Results

### Baseline characteristics and associated anomalies

All 75 patients underwent DSCT and TTE examinations without complications. The mean age was 12.68 ± 15.39 years (range: 1 month to 63 years), the mean body mass index was 17.09 ± 4.21 kg/m^2^, and the mean heart rate was 107.57 ± 27.58 bpm. A total of 103 extracardiac-vascular, 39 intracardiac, and 13 valve anomalies were confirmed. Collateral circulation (56, 74.67%), PDA (41, 54.67%), and VSD (31, 41.33%) were the most common intracardiac and extracardiac anomalies (Table 1).

**Table 1:** Characteristics in patients with CoA and IAA.

	All (n = 75)	CoA (n = 63)	IAA (n = 12)	P value
Age (y)	12.68 ± 15.39	14.36 ± 16.09	3.85 ± 5.81	0.029
Male gender	46 (61.33%)	41 (65.08%)	5 (41.67%)	0.129
BSA (m^2^)	0.89 ± 0.59	0.95 ± 0.60	0.58 ± 0.40	0.046
BMI (Kg/m^2^)	17.09 ± 4.21	17.46 ± 4.35	15.15 ± 2.82	0.082
Heart rate (bpm)	107.57 ± 27.58	106.73 ± 28.69	112.00 ± 21.29	0.548
SBP (mmHg)	119.35 ± 31.67	119.95 ± 31.95	116.17 ± 31.29	0.707
DBP (mmHg)	70.04 ± 17.23	69.92 ± 17.40	70.67 ± 17.02	0.892
HBP	31 (41.33%)	28 (44.44%)	3 (25.00%)	0.213
Extracardiac-vascular anomalies				
PDA	41 (54.67%)	29 (46.03%)	12 (100%)	0.001
APW	2 (2.67%)	0 (0.00%)	2 (16.67%)	0.001
CAA	4 (5.33%)	2 (3.17%)	2 (16.67%)	0.058
Collateral circulation	56 (74.67%)	44 (69.84%)	12 (100.0%)	0.029
Aortic valve disorders				
BAV	13 (17.33%)	11 (17.46%)	2 (16.67%)	0.947
Intracardiac anomalies				
VSD	31 (41.33%)	24 (38.10%)	7 (58.33%)	0.195
ASD	8 (10.67%)	8 (12.70%)	0 (0.00%)	0.195

Notes:Values are showed as mean ± SD or count (percent).

values < 0.05 stands for statistical significance between patients with CoA and IAA.

CoA: coarctation of aorta; IAA: interrupted aortic aorta; BSA: body surface area; BMI: body mass index; SBP: systolic blood pressure; DBP: diastolic blood pressure; HBP: high blood pressure; PDA: patent ductus arteriosus; APW: aortopulmonary window; CAA: coronary artery anomalies; BAV: bicuspid aortic valve; VSD: ventricular septal defect; ASD: atrial septal defect.

### Comparison of features between CoA and IAA

The patients in the IAA group compared to those in CoA group presented with younger age and smaller body surface area (3.85 ± 5.81 vs. 14.36 ± 16.09 years, 0.58 ± 0.40 vs. 0.95 ± 0.60 m^2^, respectively, both *p* < 0.05). PDA, aortopulmonary window, and collateral circulation were present more frequently in patients with IAA than those with CoA (100% vs. 46.03%, 16.67% vs. 0%, and 100% vs. 69.84%, respectively, all *p* < 0.05) (Table 1).

### Comparison of diagnostic accuracy between DSCT and TTE

DSCT was superior to TTE in displaying associated extracardiac-vascular anomalies (sensitivity: 100% vs. 39.81%; specificity: 100% vs. 100%; positive predictive value: 100% vs. 100%; negative predictive value: 100% vs. 76.06%). DSCT showed a similar performance in terms of TTE in aortic valve disorders (sensitivity for instance: DSCT, 61.54% vs. TTE, 61.54%) and intracardiac malformations (sensitivity for instance: DSCT, 92.31% vs. TTE, 89.74%) (Table 2).

**Table 2:** A summary of the associated cardiovascular anomalies (A) and the diagnostic accuracy (B) of DSCT and TTE (n = 75).

A	DSCT				TTE			
	TP	FN	TN	FP	TP	FN	TN	FP
Extracardiac-vascular anomalies								
PDA	41	0	34	0	38	3	34	0
APW	2	0	73	0	2	0	73	0
CAA	4	0	71	0	0	4	71	0
Collateral circulation	56	0	19	0	1	55	19	0
Aortic valve disorders								
BAV	8	5	62	0	8	5	57	5
Intracardiac anomalies								
VSD	30	1	44	0	29	2	43	1
ASD	6	2	67	0	6	2	66	1
**B**	**DSCT**				**TTE**			
	**sen**	**spec**	**ppv**	**npv**	**sen**	**spec**	**ppv**	**npv**
Extracardiac-vascular anomalies	100%	100%	100%	100%	39.81%	100%	100%	76.06%
Aortic valve disorders	61.54%	100%	100%	92.54%	61.54%	91.94%	61.54%	91.94%
Intracardiac anomalies	92.31%	100%	100%	97.37%	89.74%	98.20%	94.59%	96.46%

Notes: DSCT: dual-source computed tomography; TTE: transthoracic echocardiography; PDA: patent ductus arteriosus; APW: aortopulmonary window; CAA: coronary artery anomalies; BAV: bicuspid aortic valve; VSD: ventricular septal defect; ASD: atrial septal defect; TP: true positive finding; FN: false negative finding; TN: true negative finding; FP: false positive finding; Sen: sensitivity; Spec: specificity; PPV: positive predictive value; NPV: negative predictive value.

**Figure 1:**
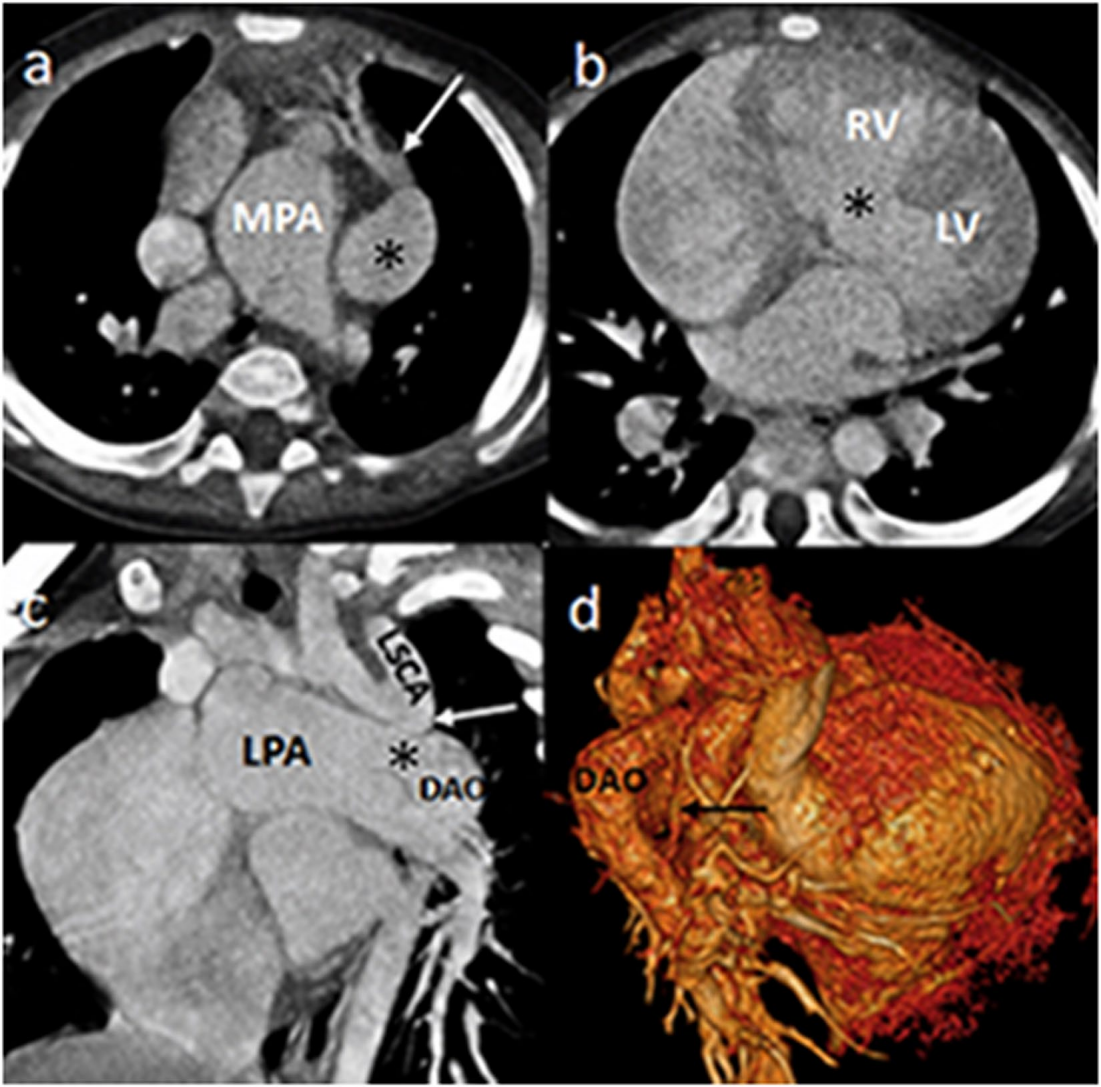
DSCT images of an 1-year-old boy with interrupted aortic aorta. The axial image (**a**) presents that the dilated right coronary aorta (white arrow) abnormally origins from the dilated left coronary sinus (asterisk) and distributes tortuously. The axial image (**b**) shows a large ventricular septal defect (asterisk). The multiplanar reformatted image (**c**) displays the aortic arch terminates at LSCA (white arrow). A ductus arteriosus (asterisk) extends from the LPA to the DAO. Volume rendering image (**d**) shows the collateral vessel (black arrow). *Abbreviations*: DSCT, dual-source computed tomography; MPA, major pulmonary artery; LV, left ventricle; RV, right ventricle; LSCA, left subclavian artery; LPA, left pulmonary artery; DAO, descending aorta.

**Figure 2:**
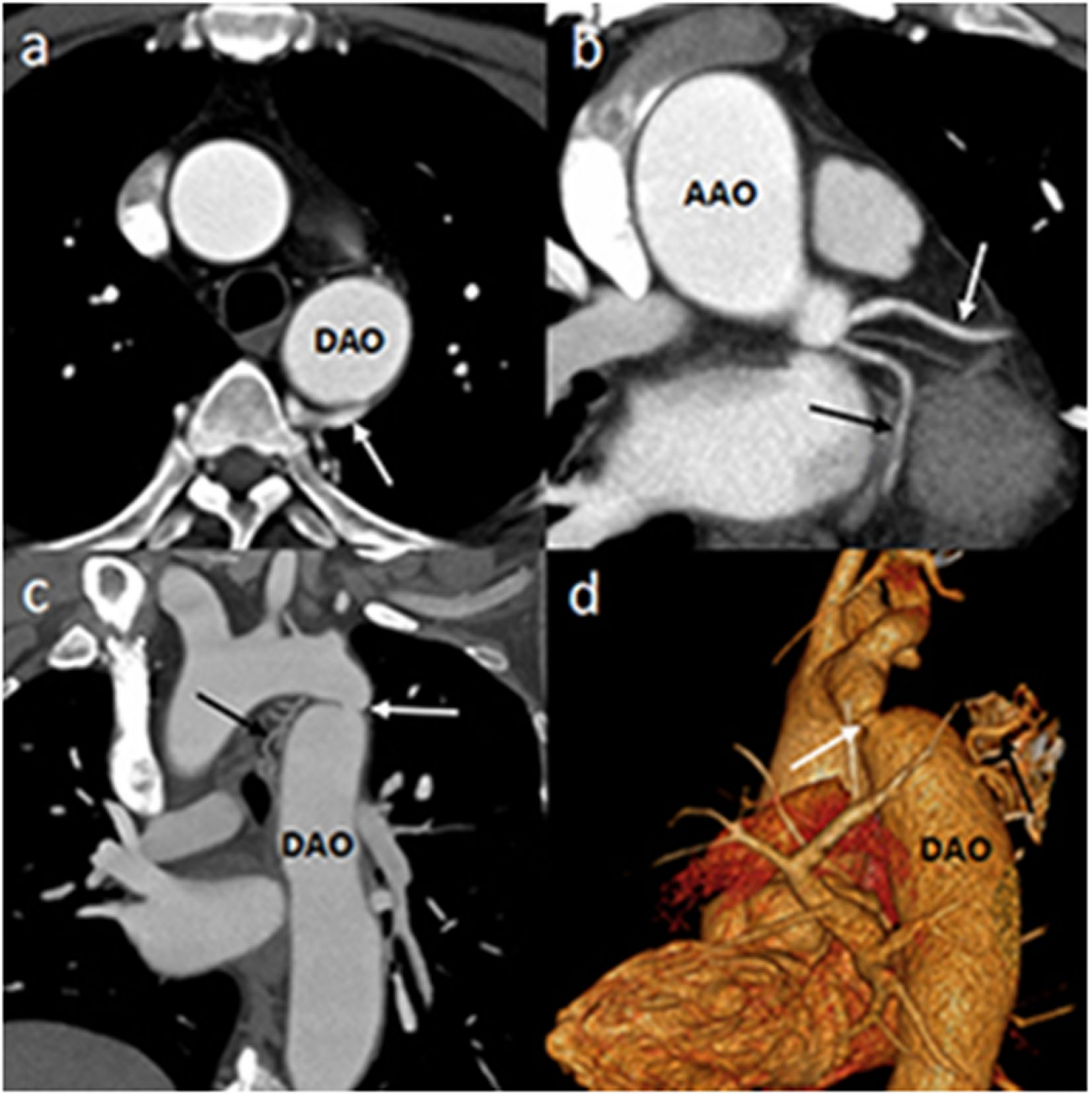
DSCT images of a 43-year-old man with coarctation of the aorta. Axial image (**a**) shows the dilated collateral vessels from DAO (white arrow). Axial multiplanar reformatted image (**b**) shows LAD (white arrow) and LCX (black arrow) origin from left coronary sinus respectively. The multiplanar reformatted image (**c**) and volume rendering image (**d**) show the site of coarctation (white arrow) and the collateral vessels (black arrow). *Abbreviations*: DSCT, dual-source computed tomography; DAO, descending aorta; AAO, ascending aorta.

Five cases of BAV and three cases of intracardiac anomalies, including one case of VSD and two of atrial septal defect, were not detected by DSCT. Conversely, TTE missed five cases of BAV, 62 cases of associated anomalies, including three of PDA, four of coronary artery anomalies, and 55 of collateral circulation, two of VSD, and two of atrial septal defect (Figs 1 and 2). In addition, TTE misdiagnosed five cases of BAV, one of VSD, and one of atrial septal defect (Table 2).

### Radiation dose estimation

The mean effective radiation dose for patients in different age groups was 0.87 ± 0.41 mSv (below four months), 1.31 ± 0.92 mSv (four months to one year), 2.23 ± 2.21 mSv (one to six years), 2.55 ± 2.44 mSv (six to ten years), and 8.29 ± 6.03 mSv (over 10 years). The mean effective dose of DSCT for patients aged < 14 years was 1.82 ± 1.82 mSv.

## Discussion

Intracardiac and extracardiac anomalies are commonly presented in patients with CoA and IAA, which influence the treatment planning and postoperative effect^1–4,13^. Thus, it is necessary to identify an appropriate imaging method to achieve a precise preoperative evaluation of these associated intracardiac and extracardiac anomalies in patients with CoA and IAA^1,14–16^.

Several methods are used to evaluate CoA and IAA. TTE, as the first-line imaging method, is limited in assessing vascular deformities and associated collateral circulation due to low spatial resolution, poor imaging window, and technician-dependent nature^17,18^. Cardiac catheterization, the gold standard for assessing the aorta, is restrained by its invasive nature in preoperative evaluation^19^. Magnetic resonance imaging, with an advantage of no radiation exposure, has been widely used in this field^20,21^. However, the relatively longer examination time, higher cost, and lower spatial resolution compared with CT restrict its application^22^. As a noninvasive imaging technology, DSCT has a high spatial resolution, wide field of view, and valuable image post-processing techniques. It is considered a reliable method to describe the aorta and associated deformities^5^.

During the period of the study, the continuously enrolled patients received both DSCT and TTE examinations as a matter of routine. Thus, we reduced the selection bias to some extent by excluded the clinician judgement. In addition, we excluded patients without surgery or cardiac catheterization finally, which was the exact reference; patients with insufficient clinical information, and other aortic diseases, or history of cardiovascular treatment. Therefore, we also decreased the possible factors that may impact the evaluation of DSCT and TTE.

Our study demonstrated that PDA and collateral circulation appeared in all patients with IAA, more commonly than in patients with CoA. It may be ascribed to that some collateral vessels must develop to act as a compensatory blood flow channel and further maintain the systemic circulation, based on the nature of discontinuity, not stenosis, between the aortic arch and descending aorta^1,4^. The relatively frequent occurrence of aortopulmonary window in patients with IAA is due to a similar factor^4^. In addition, VSD was a common intracardiac anomaly in patients with IAA and CoA, which is consistent with some previous studies, and may be attributed to the deformity between the conal septum and embryonic trunk or ventricular septum^4,23^. Some studies suggested that IAA was the extreme manifestation of CoA, with a pathogenetically close relationship^1,23^. In the current study, the incidence of aortic disorders and intracardiac anomalies is similar in two aortic congenital diseases, whereas the associated extracardiac anomalies are significantly different. Both CoA and IAA are congenital aortic arch diseases with a similar performance that are due to developmental disorders. However, the exact sites and types of variation are notably diverse, which contributes to the different extracardiac-vascular manifestations^1,12,23^. Thus, accurate preoperative differential diagnosis and evaluation of CoA and IAA are necessary to achieve comprehensive and successful treatment planning.

In our current study, DSCT demonstrated a better visualization of extracardiac-vascular malformations, including PDA, aortopulmonary window, coronary artery anomaly, and collateral circulation, than TTE. In TTE, the relatively smaller field of view from the suprasternal direction and the overlying bone and lung during examination may explain its weakness in evaluating the extracardiac vessels and associated collateral vessels. In aortic valve disorders and intracardiac anomalies, DSCT presents no significant advantage over TTE. This can be partially explained by the transfer of digital information to gray-scale images in DSCT, which makes it difficult to detect the valvular and intracardiac details^7^. Although TTE has advantages over DSCT in assessing some valvular anomalies, some combined and adjacent coronary deformities still remain difficult to identify by TTE alone^24^. With the wide application of advanced post-processing techniques, DSCT can precisely evaluate the coronary arteries^24,25^. Collateral circulation is a crucial factor in the preoperative evaluation of patients with CoA and IAA. It plays an important role in the regulation of systemic blood distribution and hemodynamic changes, further contributing to operation regimen management^1,26^. Our results show that DSCT can reliably detect combined extracardiac-vascular anomalies in patients with CoA and IAA with 100% sensitivity and specificity, which agrees with the results of a previous study^27^. In this respect, TTE shows a relatively weak value for its limited acoustic window. For CoA and IAA, DSCT can provide a more comprehensive and clearer visualization of related preoperative anatomy details, including the location, morphology, and adjacent vessel connections of abnormal aorta, which are also crucial in clinical intervention and postoperative results^12,27^. As our results show, DSCT is necessary to precisely assess extracardiac anomalies with a similar performance with TTE in evaluating the intracardiac and aortic valve disorders. Thus, combining TTE and DSCT may contribute to improve strategic planning for preoperative patients with CoA and IAA.

There were several limitations in the current study. First, given the low prevalence of CoA and IAA as well as the strict inclusion and exclusion criteria, this retrospective single-center study only acquired limited clinical and imaging data; however, the existing information was sufficient to perform the primary research. Second, although pediatric patients were exposed to some radiation from CT, DSCT was used to reduce this radiation. The mean effective dose of DSCT for patients aged <14 years was 1.82 ± 1.82 mSv, which was at a clinically acceptable level. Third, a larger multi-center study with long-term follow up is necessary to evaluate the characteristics of patients in the future.

In conclusion, collateral circulation, PDA, and VSD are the most commonly associated anomalies in patients with CoA and IAA. With similar diagnostic performances of TTE in evaluating intracardiac and aortic valve anomalies, DSCT can reliably provide a more overall preoperative evaluation of associated extracardiac-vascular malformations, with anatomical details including local aortic lesion and adjacent collateral vessels for surgical decisions.

## Material and Methods

### Study population

A total of 138 patients with CoA or IAA who were referred to our hospital between August 2010 and June 2018 were retrospectively studied. The inclusion criteria were patients who underwent preoperative DSCT and TTE, as well as patients with surgery or cardiac catheterization indication. The exclusion criteria were patients without surgery or cardiac catheterization finally (27 cases), with insufficient clinical information(23 cases), other aortic diseases, such as aortic arch hypoplasia, atherosclerosis, arteritis, malformations like Turner syndrome and Marfan syndrome, or history of cardiovascular catheter or surgical interventions (13 cases). The current research was conducted in accordance with the ACC/AHA 2008 Guidelines^28^. Finally, 75 patients remained (63 with CoA and 12 with IAA; 46 men and 29 women). The institutional review board of West China Hospital of Sichuan University approved our study (No. 14–163), and we declared to comply with the declaration of Helsinki (2000 EDITION) and associated research rules of China in the current study. Before CT scans, all patients or their guardians signed informed consent about radiation exposure and potential adverse reactions to iodinated contrast medium. Patient-sensitive information was kept full confidentially and only used for the current study. The names of participants and other HIPAA identifiers had been removed from all sections of the manuscript.

### Scanning protocol

DSCT was performed with a scanner (Somatom Definition; Siemens Medical Solutions, Forchheim, Germany), and a retrospectively ECG-gated protocol was used with the following acquisition parameters: tube current of 100–220 mAs, tube voltage of 80–120 kV, pitch of 0.2–0.5, and gantry rotation time of 0.28–0.33 s. A short-term sedative (chloral hydrate with 10% concentration, 0.5 mL/kg) was used before the cardiac DSCT examinations for patients younger than 6 years old. When scanning, older patients were asked to hold the breath. In the craniocaudal direction, the scan was performed from the inlet of the thorax to 2 cm below the level of the diaphragm. All patients was injected to nonionic contrast agent (iopamidol, 370 mg/mL, Bracco, Italy) by an antecubital vein, with a rate of 1.2–2.5 mL/s, and then 20 ml of saline solution was injected. The injected volume referred to the body weight (1.5 ml/kg). Bolus tracking was used over the region of interest (ROI) of descending aorta, with a set threshold of 100 HU. Image acquisition was triggered after a delay of 5 s when the ROI attenuation threshold reached 100 HU. We analyzed all imaging data on a workstation (Syngo; Siemens Medical System, Forchheim, Germany). All images were reconstructed with 0.75-mm slice thickness and 0.70-mm increment.

### Transthoracic echocardiography

All patients conducted TTE examination with a ultrasound system (iE33; Philips Medical Systems NA, Bothell, WA, USA), via S5-1 probe (Frequency of 1–5 MHz). According to the recommendations of the American Society of Echocardiography Committee, M-mode, two-dimensional, continuous wave and Doppler color flow imaging were used^29^. The detailed information of aorta and heart was evaluated through parasternal left ventricular long axis section, short axis section of aorta, and long axis section of left and right ventricle.

### Imaging analysis

Concomitant intracardiac and extracardiac anomalies were recorded. Two experienced radiologists in a blind method used axial CT images and post-processing techniques, including multiplanar reconstruction, maximum intensity projection, and volume rendering, to analyze images. After acquiring axial CT images and their post-processing images, the two experienced radiologists analyzed the images according to the ACC/AHA 2008 Guidelines and their rich experience in department of radiology. For the analysis reproducibility, one experienced radiologist firstly completed measurements for all the images and repeated the analysis about a week later. Another experienced radiologist, who was unaware of above results, remeasured the images. If disagreement occurred, the two radiologists negotiated and reached a consensus about the data. TTE images were also assessed by two experienced cardiologists who were blinded to the results of DSCT and surgical and cardiac catheterization. The process of TTE analysis for two cardiologists is consistent of CT.

To compare the diagnostic value of DSCT with that of TTE, all the confirmed associated anomalies were classified into three groups, including extracardiac-vascular anomalies, aortic valve disorders, and intracardiac anomalies. Results of surgery or cardiac catheterization were considered as the reference standard to identify the diagnostic accuracy of both methods.

### Radiation dose estimation

Volume CT dose index and dose-length product were automatically transmitted and recorded to the CT console after DSCT examination. Based on the 2007 recommendations of the International Commission on Radiological Protection, we used conversion coefficients to calculate the effective dose^30,31^.

### Statistical analysis

 We performed statistical analysis by SPSS software for Windows (version 24.0, SPSS Inc., Chicago, IL, USA). Continuous variables were presented as mean ± standard deviations and categorical variables were shown as numbers and  percentages. Kolmogorov–Smirnov test was used to test normality. Independent Student’s t-test was used to compare the continuous variables, and Mann–Whitney U test was performed to analyze the qualitative variables between two groups of aortic defects. The sensitivity, specificity, positive predictive value, and negative predictive value were used to delineate the diagnostic accuracy of DSCT and TTE for the malformations in each group. A two-tailed *p* value < 0.05 was regarded as statistical significance.

## Data Availability

The datasets generated during and/or analysed during the current study are available from the corresponding author on reasonable request.
